# Short and Long Term Effects of Left and Bilateral Repetitive Transcranial Magnetic Stimulation in Schizophrenia Patients with Auditory Verbal Hallucinations: A Randomized Controlled Trial

**DOI:** 10.1371/journal.pone.0108828

**Published:** 2014-10-20

**Authors:** Leonie Bais, Ans Vercammen, Roy Stewart, Frank van Es, Bert Visser, André Aleman, Henderikus Knegtering

**Affiliations:** 1 University of Groningen, University Medical Center Groningen, Department of Neuroscience and BCN NeuroImaging Center, Groningen, The Netherlands; 2 Lentis Psychiatric Institute, Groningen, The Netherlands; 3 Australian Catholic University, Strathfield, Australia; 4 University of Groningen, University Medical Center Groningen, Department of Health Sciences, Community and Occupational Medicine, Groningen, The Netherlands; 5 University of Groningen, University Medical Center Groningen, Department of Psychiatry, Groningen, The Netherlands; 6 University of Groningen, Department of Psychology, Groningen, The Netherlands; 7 University of Groningen, University Medical Center Groningen, Rob Giel Research Center, Groningen, The Netherlands; Osaka University Graduate School of Medicine, Japan

## Abstract

**Background:**

Repetitive transcranial magnetic stimulation of the left temporo-parietal junction area has been studied as a treatment option for auditory verbal hallucinations. Although the right temporo-parietal junction area has also shown involvement in the genesis of auditory verbal hallucinations, no studies have used bilateral stimulation. Moreover, little is known about durability effects. We studied the short and long term effects of 1 Hz treatment of the left temporo-parietal junction area in schizophrenia patients with persistent auditory verbal hallucinations, compared to sham stimulation, and added an extra treatment arm of bilateral TPJ area stimulation.

**Methods:**

In this randomized controlled trial, 51 patients diagnosed with schizophrenia and persistent auditory verbal hallucinations were randomly allocated to treatment of the left or bilateral temporo-parietal junction area or sham treatment. Patients were treated for six days, twice daily for 20 minutes. Short term efficacy was measured with the Positive and Negative Syndrome Scale (PANSS), the Auditory Hallucinations Rating Scale (AHRS), and the Positive and Negative Affect Scale (PANAS). We included follow-up measures with the AHRS and PANAS at four weeks and three months.

**Results:**

The interaction between time and treatment for Hallucination item P3 of the PANSS showed a trend for significance, caused by a small reduction of scores in the left group. Although self-reported hallucination scores, as measured with the AHRS and PANAS, decreased significantly during the trial period, there were no differences between the three treatment groups.

**Conclusion:**

We did not find convincing evidence for the efficacy of left-sided rTMS, compared to sham rTMS. Moreover, bilateral rTMS was not superior over left rTMS or sham in improving AVH. Optimizing treatment parameters may result in stronger evidence for the efficacy of rTMS treatment of AVH. Moreover, future research should consider investigating factors predicting individual response.

**Trial Registration:**

Dutch Trial Register NTR1813

## Introduction

About 50–70% of the patients fulfilling the criteria for schizophrenia experience auditory verbal hallucinations (AVH) on a frequent basis [1]. Antipsychotic medication is often chosen as the first treatment for this disabling symptom of schizophrenia. However, in 25% of the patients, hallucinations appear to be refractory to adequate treatment trials with antipsychotic medication [2]. The high level of burden accompanied with AVH urges for the development of more efficient treatments.

Understanding the underlying neural basis of AVH may be helpful in the development of better treatment options. AVH have been related to both structural and functional anomalies in frontal, temporal and parietal regions [3], [4]. Furthermore, cingular, subcortical and cerebellar areas have shown involvement as well (for an overview, see: [5]). Several theories about the genesis of AVH have been suggested, but the most popular theory argues that AVH are a result of misattributions of inner speech to an external source [6]. Allen et al. [5] postulated a neurocognitive model in which they state that over-activation of primary and secondary auditory cortices may be associated with bottom-up over-perceptualization. Meanwhile, aberrant activation in Broca’s area (speech production), and reduced connectivity between Broca’s area and the anterior cingulate (monitoring) and Wernicke’s area (speech reception), may lead to reduced top-down control, ultimately resulting in the experience of auditory verbal hallucinations.

Given the hyperactivation within speech perception areas during the experience of AVH, it seems plausible that modulation of the neuronal activity could reduce AVH. The left temporo-parietal junction area (TPJ) is strongly connected with primary and secondary auditory cortices, and has therefore been proposed as a target region for treatment with low frequency repetitive Transcranial Magnetic Stimulation (rTMS). rTMS constitutes the administration of brief magnetic pulses with an electro-magnetic coil on the scalp. Low frequency rTMS can reduce cortical excitability within the underlying cortical tissue and connected deeper brain tissues in healthy volunteers [7]–[9]. Although the exact mechanism of action is not yet understood, the effect of low frequency rTMS is being explained in terms of long-term depression (LTD)-like changes in synaptic efficacy [10]. In rats, it has been shown that the inhibiting effect on cortical excitability endures after multiple rTMS sessions [11]. Researchers have therefore begun to apply this technique in the treatment of neurological and psychiatric disorders [12] associated with aberrant cortical activity. Hoffman et al. [13], [14] pioneered this intervention in schizophrenia patients experiencing AVH. Since the early 2000’s a number of rTMS trials have been initiated, aiming to treat AVH, with the majority targeting the left TPJ area [15]–[31]. Recent basic cognitive rTMS studies have shown the relevance of the TPJ area in the language and AVH network [32]–[36].

Meta-analytical reviews of the treatment studies have shown moderate to large effect sizes [37]–[39]. However, the majority of these effect sizes are based on the severity of symptoms immediately after treatment. Little is known about the duration effects of rTMS for AVH. Only a few studies have been published on the effects after more than four weeks [16], [19], [20], [28]. From a clinical perspective, insight into long-term effects of this type of treatment would be highly advantageous.

Despite neuroimaging findings on the involvement of both the left and right temporal cortex in the genesis of AVH [3], [4], most treatments have restricted stimulation of low frequency rTMS to the left TPJ area. This treatment has been found to influence characteristics like frequency and attentional salience of AVH [15], [16]. The right hemisphere is considered to be dominant for the processing of emotions [40], especially negative emotions [41]. As AVH are often negative in emotional content [42], [43], it is conceivable that low frequency rTMS treatment over the right hemisphere down-regulates this negative content.

As an extension of the trial reported by Vercammen et al. [44], we report on a randomized controlled trial in schizophrenia patients with 1 Hz rTMS over the left TPJ area, compared to sham stimulation over the left TPJ area. To investigate whether stimulation over the right TPJ area would have an additional effect, we added an extra treatment arm of bilateral TPJ area stimulation. We included follow-up measures at four weeks and three months after treatment. We hypothesized that 1 Hz rTMS over the left TPJ area would result in improvement of AVH, compared to sham stimulation, and that reduction of AVH would be further enhanced after bilateral stimulation, compared to both left TPJ stimulation and sham.

## Materials and Methods

The protocol for this trial and supporting CONSORT checklist are available as supporting information; see Checklist S1 and Protocol S1.

### Participants

From July 2006 to March 2012, patients were recruited at University Medical Center Groningen (UMCG), and collaborating regional mental health foundations: Lentis, GGz Friesland, GGz Drenthe and Mediant. All patients met DSM-IV criteria for schizophrenia; diagnoses were confirmed using Schedules for Clinical Assessment in Neuropsychiatry [45]. Only patients reporting frequent (at least daily) medication resistant AVH were included. Medication resistance was defined as daily occurring AVH despite at least two adequate trials of antipsychotic medication for at least four weeks prior to study inclusion. Medication dose remained unchanged for the duration of the study. To minimize the risk of inducing seizures by rTMS, patients with a personal or family history of epileptic seizures were excluded. Other exclusion criteria were: a history of severe head trauma or neurological disorder, the presence of intra-cerebral or pacemaker implants, inner ear prosthesis or other metal prosthetics/implants, severe behavioral disorder, current substance abuse, and pregnancy.

This study was approved by the licensed local medical ethical committee of the University Medical Center Groningen (number 2006/052) and conducted in accordance with the latest version of the Declaration of Helsinki (Protocol S1). As it was not yet customary in The Netherlands to register non-pharmaceutical trials when our trial started in 2006, we registered the trial in 2009 in the Dutch trial register when it was advised to do so (www.trialregister.nl, number: NTR1813). The authors confirm that all ongoing and related trials for this intervention are registered. Only patients who were competent to give informed consent were included. Prior to the study, all subjects gave oral and written informed consent after the procedure had been fully explained. All obtained subject data was handled anonymously.

This study was a double-blind randomized controlled trial. Treatments were given in the University Center of Psychiatry of the UMCG. Before the start of the trial, envelopes were numbered sequentially with participant ID numbers. An independent colleague drew tokens for one of the three treatment conditions, which were subsequently put into the envelopes. The envelopes were sealed, and prior to the start of each participant’s treatment period, the researcher (AV or LB) opened the envelope with the number that corresponded to the participant’s allocated ID number. Participant ID numbers were allocated consecutively based on date of participant intake, which was not controlled by the TMS administrator. Only the persons who administered the TMS (either the researchers or a trained nurse) were aware of the treatment condition. All other people involved – participants, clinical raters and clinicians – were kept blind. To check for blinding success, the participants completed a questionnaire. Participants were informed about the received treatment when the last follow-up measurement at three months was completed.

During the trial, patients were either admitted to an inpatient care unit, a day-hospital, or visited the hospital twice a day. Demographic and clinical characteristics at baseline are provided in Table 1.

**Table 1:** Demographic data and baseline characteristics of the sample.

		Left TMS (n = 16)	Bilateral TMS (n = 15)	Sham TMS (n = 16)	p
Age (years)	Mean (+/− SD)	37.2 (14.9)	33.9 (9.2)	37.3 (11.6)	0.688
Sex (m/f)	N	9/7	8/7	10/6	0.869
Education (years)	Mean (+/− SD)	13.7 (2.0)	13.4 (1.2)	13.7 (2.1)	0.870
Age of onset (years)	Mean (+/− SD)	26.4 (10.2)	23.1 (4.2)	21.8 (6.9)	0.230
Duration of illness (months)	Mean (+/− SD)	150.4 (123.2)	135.8 (123.4)	186.7 (149.4)	0.559
Type of medication					
*Clozapine*		6	7	6	
*Olanzapine*		4	2	4	
*Risperidone*		3	3	3	
*Aripiprazole*		3	1	3	
*Quetiapine*		2	3	1	
*Sulpiride*		–	–	1	
*Haloperidol*		–	–	3	
*Other first generation*		1	3	4	
*None*		1	–	1	
Motor threshold	Mean (+/− SD)	60.2 (7.4)	56.7 (9.5)	–	0.366
AHRS frequency item	Mean (+/− SD)	6.9 (2.8)	5.9 (2.7)	5.9 (3.0)	0.521
AHRS total	Mean (+/− SD)	28.3 (5.7)	25.6 (6.7)	24.8 (6.0)	0.241
PANSS P3 (Hallucinations)	Mean (+/− SD)	5.2 (0.7)	4.6 (0.6)	4.7 (0.7)	0.036
PANSS Positive	Mean (+/− SD)	16.3 (4.8)	15.8 (3.9)	16.7 (4.6)	0.856
PANSS Negative	Mean (+/− SD)	15.1 (4.7)	13.7 (4.7)	16.6 (5.6)	0.269
PANSS General					
Psychopathology	Mean (+/− SD)	30.1 (8.9)	27.7 (6.2)	32.4 (9.5)	0.300

M = male; F = female; AHRS = Auditory Hallucinations Rating Scale [20]; PANSS = Positive and Negative Syndrome Scale [24]; PANAS = Positive and Negative Affect Scale [50]. Groups were compared using analysis of variance (ANOVA), followed by Bonferroni’s post-hoc tests in case of significant effects. The chi square test was applied to test group differences on the nominal variable sex.

### rTMS procedure

We used a Magstim Rapid System (Magstim Company Ltd, Whitland, Wales) with a 70 mm figure-of-eight coil. Sham stimulation was administered using a coil that produced the same clicking sound, without delivering a measurable magnetic field. Before the first treatment session, resting motor threshold was only determined in patients enrolled in an active treatment condition. The resting motor threshold is defined as the minimum intensity to induce a noticeable movement of the dominant hand in five out of ten pulses administered on the contralateral primary motor cortex [46]. We did not determine the motor threshold in the patients that were to receive sham treatment, to reduce the risk of unblinding the participant to the treatment condition, as our sham procedure produces no physical sensation, but the motor threshold determination does.

The localization of the TPJ area was based on the 10–20 International System of EEG electrode positions. Both active and sham stimulation of the left TPJ area were administered halfway between T3 and P3 electrode positions. In the bilateral condition the left TPJ area was stimulated for the first 10 minutes of the session, after which the coil position was switched to the right TPJ area, halfway between T4 and P4 electrode positions. The 10–20 system has been commonly used in previous rTMS trials for AVH [16], [19]–[24], [26], [27], [47], because it is a patient-friendly compromise between time efficiency and individual variability, as it takes individual head size into account [48].

Treatment was conducted for six consecutive days (except during the weekends), twice daily, for 20 minutes at 1 Hz on 90% of resting motor threshold. Patients thus received a total of 14400 pulses during the treatment. There was always a minimum period of five hours between two treatment sessions.

### Power calculation

Previous research using a similar design and rTMS parameters [22] found significant effects of the treatment on behavioral variables (Auditory Hallucination Rating Scale). An effect size of 1.22 was observed [22], [37]. With the inclusion of 16 subjects in each treatment arm, and an alpha of <.05, a power of.90 would be acquired.

### Outcome parameters

All participants were interviewed with the semi-structured Positive and Negative Syndrome Scale (PANSS) [49] before and immediately after treatment by two trained raters. For the analyses we used the scores on hallucination item P3, as well as scores on the subscales for Positive symptoms, Negative symptoms and General Psychopathology. Characteristics of the AVH were measured with the Auditory Hallucinations Rating Scale (AHRS), which is a self-report questionnaire [15]. It comprises seven items (frequency, reality, loudness, number of voices, length, attentional salience, distress level). AHRS frequency scores and total AHRS scores were used for the analysis. Positive and negative emotional affective states were measured with the Positive and Negative Affect Scale (PANAS) [50] adapted for hallucinations (note that this is a different scale than the PANSS). Participants were instructed to indicate to what extent they experienced ten positive and ten negative affective states during AVH. Items had to be rated on a five-point scale, ranging from 1 = very slightly to 5 = extremely. Sum scores of all positive and all negative items were calculated and used for analysis. Both the AHRS and PANAS were administered before and immediately after treatment. For the measurements at four weeks and three months follow-up we sent the questionnaires by mail to the participants.

### fMRI procedure

In 36 participants, fMRI scans were made before and after rTMS treatment, the results of the fMRI analyses are discussed elsewhere [51].

### Statistics

Differences in demographic characteristics and baseline data between the three treatment groups were tested with analysis of variance (ANOVA), followed by Bonferroni’s post-hoc tests in case of significant effects. Chi-square tests were applied to the nominal variables.

The influence of patients, time, and treatment on the outcome measures of the PANSS (Hallucination item P3, Positive symptoms, Negative symptoms and General Psychopathology), AHRS (Frequency and Total) and PANAS (Positive and Negative) were analyzed using multilevel modeling, because this technique handles missing data better than single level designs. It does not require balanced designs (e.g. same sample sizes in each level of the design) and is more flexible.

A two-level random intercept model was fitted to the data. Repeated measurements in Time (for the PANSS: baseline and end of treatment; for the AHRS and PANAS: baseline, end of treatment, four weeks follow-up and three months follow-up) were nested within patients. Group (left rTMS, bilateral rTMS and sham rTMS) was added as a covariate. The main effects of Time and Group are given, as well as their interactions. In case of significant main or interaction effects, estimates of fixed effects are presented, to clarify which differences caused the effects. P-values of <.05 were considered statistically significant. We used the IBM software package SPSS version 21 to fit the multilevel model to the data.

## Results

### Participants

**Figure 1:**
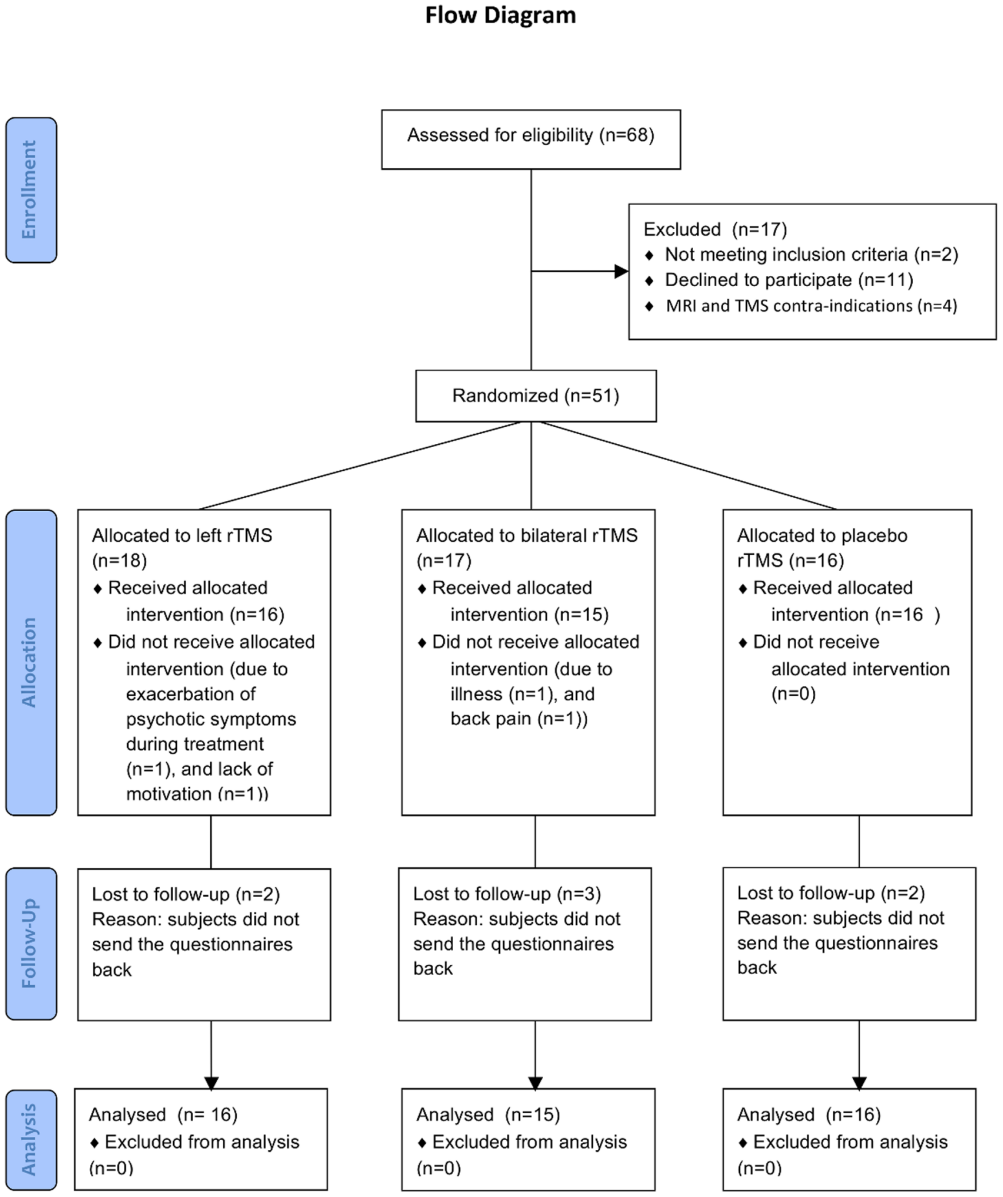
Flow diagram demonstrating the progress of the trial.

The flow diagram (Figure 1) demonstrates the progress of the trial. 68 patients were assessed for eligibility, and eventually 51 patients were included in the trial. A total of four patients withdrew from the study, for various reasons, which are described in the safety and tolerability section. So, 47 participants completed the study, with 16 patients in the left group, 15 patients in the bilateral group, and 16 patients in the sham group. Demographic characteristics did not differ significantly between the three treatment groups (Table 1). There were no baseline differences between groups in hallucination severity as reflected by the AHRS. Baseline scores on hallucination item P3 of the PANSS were not equal between the three groups; however post-hoc testing revealed no significant differences between the groups (Table 1).

### Short term-efficacy measured with the PANSS P3 item and subscales

**Figure 2:**
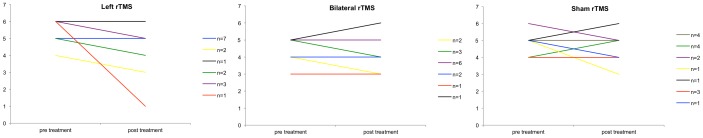
Changes in PANSS hallucination scores. This figure illustrates the individual courses in hallucinations score. Each line represents how many subjects showed a specific change in hallucination score, separate for the three treatment groups.

Mean scores on the PANSS are displayed in Table 2. There was no significant main effect of group on the PANSS Hallucination item P3 (F(2,44.0) = 1.034, p = 0.364). However, the main effect of time (pre- vs. post-treatment) was significant (F(1,44.0) = 5.942, p = 0.019). The interaction between time and treatment group showed a trend for significance (F(2,44.0) = 2.545, p = 0.090), which was caused by a small decrease of hallucination severity in the left treatment group. Figure 2 illustrates the individual courses in hallucination scores. Each line represents how many subjects showed a specific change in hallucination score, separate for the three treatment groups.

**Table 2:** PANSS, AHRS and PANAS scores at all four measurement moments.

		Baseline			End of treatment			4 weeks follow-up			3 months follow-up		
	Group	mean	SD	n	mean	SD	n	mean	SD	n	mean	SD	n
PANSS	Left	5.19	0.66	16	4.44	1.21	16	–			–		
Item P3	Bilateral	4.60	0.63	15	4.33	0.90	15	–			–		
	Sham	4.69	0.70	16	4.69	0.70	16	–			–		
PANSS	Left	16.31	4.76	16	15.06	5.64	16	–			–		
Positive	Bilateral	15.80	3.88	15	15.21	4.14	15	–			–		
	Sham	16.69	4.60	16	16.56	3.88	16	–			–		
PANSS	Left	15.12	4.70	16	14.50	4.40	16	–			–		
Negative	Bilateral	13.67	4.67	15	14.00	4.95	15	–			–		
	Sham	16.63	5.57	16	16.81	5.04	16	–			–		
PANSS	Left	30.12	8.85	16	28.38	9.04	16	–			–		
General	Bilateral	27.67	6.20	15	26.71	5.81	15	–			–		
	Sham	32.50	9.41	16	31.56	7.50	16	–			–		
AHRS Frequency	Left	6.88	2.83	16	5.50	3.06	16	5.07	3.13	14	5.14	3.18	14
Frequency item	Bilateral	5.87	2.70	15	5.13	3.07	15	5.83	3.19	12	5.42	3.23	12
	Sham	5.88	2.96	16	4.75	3.00	16	4.14	2.91	14	4.14	2.83	14
AHRS	Left	28.31	5.67	16	26.13	5.55	16	24.79	8.76	14	24.29	9.43	12
Total	Bilateral	25.60	6.73	15	23.27	7.09	15	22.50	8.10	12	23.92	7.10	14
	Sham	24.75	5.97	16	21.63	9.95	16	20.00	10.41	14	21.79	9.41	14
PANAS	Left	28.79	10.60	14	24.47	9.67	15	24.33	10.34	15	24.19	10.1	16
Positive	Bilateral	25.67	10.10	15	19.93	8.08	15	21.53	7.92	15	22.13	7.99	15
	Sham	21.08	6.97	14	19.14	7.16	14	17.69	6.86	13	17.62	6.20	13
PANAS	Left	21.50	7.98	14	20.13	8.40	15	18.27	6.72	15	18.94	8.44	16
Negative	Bilateral	23.40	10.53	15	21.80	11.20	15	19.40	9.42	15	19.53	9.14	15
	Sham	29.43	10.30	13	25.43	10.60	14	24.92	11.93	13	26.46	13.0	13

Means, standardg deviations and the number of participants in each measurement are presented for the three treatment groups. AHRS = Auditory Hallucinations Rating Scale [20]; PANSS = Positive and Negative Syndrome Scale [24]; PANAS = Positive and Negative Affect Scale [50].

Treatment group or time did not have significant effects on the total positive symptoms, negative symptoms and general psychopathology subscales.

### Short term and long term efficacy, as measured with the AHRS

**Figure 3:**
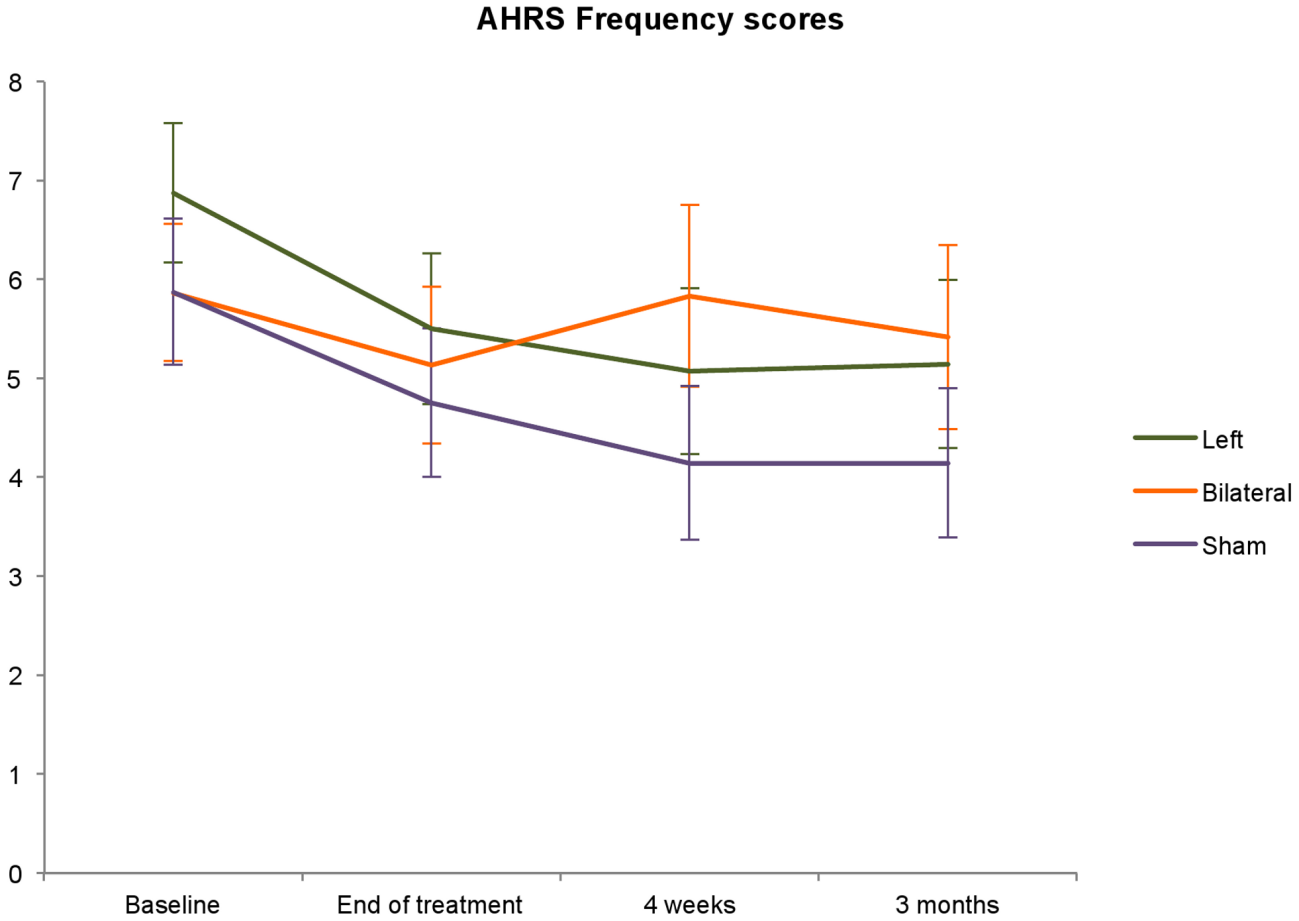
Changes in AHRS Frequency scores. The three lines represent the mean AHRS Frequency scores for each treatment group during the four measurements at baseline, end of treatment, and at four weeks and three months follow-up.

Mean scores on the AHRS are displayed in Figure 3 and Table 2. Analysis revealed a significant main effect of the factor time for the AHRS Frequency scores (F(3,41.6) = 4.92, p = 0.005). Table 3 summarizes the results of pairwise comparisons and shows that the means on all post treatment measurements decreased significantly compared to baseline. So AHRS frequency scores were significantly lower at the end of treatment, when compared to the measurement at baseline and remained on a lower level for at least three months (Table 3). The reduction of AHRS frequency scores over time was independent of the treatment condition, as there was no main effect of treatment, or interaction between time and treatment.

**Table 3:** Pairwise comparisons between post-treatment measurements and baseline.

	AHRS Frequency	AHRS Total	PANAS Positive	PANAS Negative
	Mean difference	Mean difference	Mean difference	Mean difference
End of Treatment - Baseline	1.08**	2.55	4.57**	1.85
4 weeks follow-up - Baseline	1.26**	3.35*	4.77**	3.63**
3 months follow-up - Baseline	1.31**	3.03*	4.04**	3.61**

**significant at p≤0.05. *trend for significance: 0.01<p<0.05. AHRS = Auditory Hallucinations Rating Scale [20]; PANSS = Positive and Negative Syndrome Scale [24]; PANAS = Positive and Negative Affect Scale [50].

Total AHRS scores also decreased over time (F(3,40.9) = 2.89, p = 0.047). However, pairwise comparisons only showed trends for significance between the mean at baseline and the means at four weeks follow-up and three months follow-up (Table 3). Again, main effects of treatment and the interaction between treatment and time were not significant.

### Short term and long term efficacy, as measured with the PANAS

Mean scores on the PANAS are displayed in Table 2. Both negative and positive affect scores as measured with the PANAS showed significant decreases in time (F(3,38.0) = 5.69, p = 0.003; F(3,40.5) = 6.29, p<0.001, respectively). In case of the PANAS negative scores, this effect was caused by significant decreases after four weeks follow-up compared to baseline, and remained decreased until three months follow-up. The PANAS positive scores decreased during the treatment and stayed on a constant level for three months (Table 3). There were no main effects for treatment or interaction effects on either PANAS positive or negative scores.

### Responder analysis

Eight patients in the left group improved one point or more on the PANSS Hallucination item P3, against five patients in the bilateral group and four in the sham group. In five patients from the sham group, the scores increased one point, against one patient in the bilateral group and none in the left group.

When considering a reduction of three points or more on the AHRS frequency item as a response, five patients in the left group were responders, and one patient in the bilateral group, against three in the sham group.

### Blinding

Not all data on blinding success were available. Out of the sixteen participants surveyed in the left group, ten thought they received real treatment (62.5%), in the bilateral group nine out of twelve surveyed thought they received real treatment (75%), against ten out of sixteen patients surveyed in the sham group that believed they received sham rTMS (62.5%).

### TMS safety and tolerability

Four patients withdrew from the study, for the following reasons: increase of psychotic symptoms; severe back pain that the patient attributed to the rTMS treatment; and inability to visit the hospital due to illness.

In the patients that completed the treatment, rTMS was well tolerated and no serious adverse events occurred. Side-effects reported in the active rTMS groups were: twitching facial muscles (ten patients), light-headedness (one patient), earache (one patient), tingling sensation in the arm on the contralateral side of rTMS stimulation, and minor pain in the left arm (one patient). These side-effects were confined to the actual stimulation session, and disappeared immediately after stimulation. One patient that received active rTMS experienced restless legs during stimulation, but she already had these complaints before participating in the trial. Nine participants in the active groups and two in the sham group reported mild transient headache following (at least one) stimulation session. Other side-effects reported in the sham group were a tingling sensation near the ear (one patient) and blunted affect (one patient) during the stimulation session. One patient in the sham group reported concentration problems during the treatment period.

## Discussion

In this trial we randomized 51 schizophrenia patients with treatment resistant auditory verbal hallucinations to receive 1 Hz rTMS treatment of the left or bilateral TPJ area, or sham rTMS. The present study was conducted as an extension of the RCT reported by Vercammen et al. [44] on 36 patients. This is the first study on hallucinations that included an extra treatment arm of bilateral stimulation to allow for the comparison of different treatment configurations. We hypothesized that stimulation of the left TPJ area would result in decreased frequency, loudness or attentional salience of AVH through diminution of left-hemispheric hyperactivation, and that bilateral stimulation would additionally reduce affect-based aspects of AVH thought to originate in the right hemisphere. This would theoretically result in a more complete management of AVH. We observed a significant decrease in mean hallucination score that remained stable for three months. Although left-sided rTMS showed a trend to significance on the PANSS, neither left nor bilateral rTMS treatment was superior over sham treatment in reducing hallucination severity as measured with the AHRS and PANAS.

As the durability of rTMS effects is of specific interest to clinicians, we included two follow-up measurements with the AHRS and PANAS, at four weeks and three months. At each time point a reduction of AVH was found, but it was irrespective of treatment condition. This corroborates findings of other studies that included long-term follow-up assessments [20], [24], [28]. It is remarkable that hallucination severity tends to decrease over time in a relatively stable and chronically ill sample, both after active and sham treatment. It is conceivable that participating in an rTMS trial to reduce auditory verbal hallucinations, promotes awareness and a realization that AVH are a product of ‘abnormal’ neural processes, residing in the brain, rather than originating from some external source. From this point of view, it may become easier for patients to cope with their hallucinations and/or they may even find a way to influence them.

By stimulating bilaterally, we applied an unconventional approach in the study of rTMS for AVH. Three previous studies compared the effects of right-sided rTMS with left-sided rTMS and sham stimulation, but did not observe superior effects of right-sided stimulation [21], [25], [26]. Contrary to our study, the rationale for adding a third treatment arm of right-sided rTMS in these former studies was not specifically based on the hypothesis that the right hemisphere might influence the emotional states accompanied with the AVH. We assessed positive and negative emotional states associated with the experience of hallucinations, observing an overall decrease in the scores of both positive and negative emotional states during the study period in all three treatment groups. Thus, our hypothesis that low-frequency bilateral stimulation specifically would result in more complete management of AVH could not be confirmed. The possibility should be considered that the lack of beneficial effects of bilateral stimulation is due to a neurophysiological process referred to as transcallosal inhibition. Thiel et al. [52] have shown that low frequency rTMS of the left inferior frontal gyrus resulted in increased activity in the right inferior frontal gyrus. In our design, inhibition of the left TPJ area might have caused disinhibition of its corresponding contralateral area, and vice versa, which may have produced a null-effect. Theoretically, low frequency rTMS of the left TPJ area, combined with high frequency rTMS of the right TPJ area, may induce a stronger effect. As various bilateral approaches are possible, further exploratory work will be necessary to clarify which configuration would be most advantageous in the treatment of hallucinations. Another explanation for the lack of improvement in the bilateral condition may be that each hemisphere received 50% fewer rTMS pulses than in the left rTMS condition, such that the total amount of pulses applied to participants was equal in every treatment condition. Perhaps 20 minutes of 1 Hz rTMS on each side, might have achieved a significant treatment effect.

Nevertheless, as we did not observe improvement in either of the active rTMS conditions, it may be more plausible that the overall lack of substantial, clinically relevant improvements can be ascribed to methodological variables. Increasing stimulation intensity, varying frequency, and extending duration of treatment may positively influence efficacy. For instance, a two-day high-frequency treatment has demonstrated remarkable efficacy in two studies [47], [53]. Moreover, it is still debatable whether the left TPJ area – which is assumed to be hyperactive – should be stimulated in isolation. Recent imaging findings revealed reduced connectivity and information flow between left frontal and temporal areas in schizophrenia patients with AVH [54]–[56]. Additionally, hypofrontality has been reported in schizophrenia patients [57], [58]. Taking this into account, high-frequency rTMS over the left frontal area combined with low-frequency rTMS over the left TPJ area, may strengthen fronto-temporal pathways, potentially resulting in a more efficacious reduction of hallucination severity. To our knowledge, this type of stimulation has not been performed yet with rTMS. However, Brunelin et al. [59] obtained interesting results using transcranial Direct Current Stimulation (tDCS). With tDCS, cathodal and anodal electrodes are applied, which respectively result in decreased or increased membrane potentials of neurons underlying the respective stimulation sites. Cathodal left TPJ area stimulation to decrease underlying tissue activity and anodal left dorsolateral prefrontal stimulation to elicit an increase in activity resulted in a substantial decrease in AVH after 5 days. In addition, this improvement sustained for at least three months in the treatment group, whereas the sham group did not show significant improvement. It goes without saying that replication studies are required to substantiate this initial finding [60].

Aside from variability in stimulation parameters, variability between subjects in response to rTMS treatment should also be taken into account. Although not clinically significant, responder analysis demonstrated that in the left rTMS group, twice as many patients improved at least one point on the PANSS hallucination item compared to the sham group. This finding might suggest that left-sided rTMS does have some effect on AVH. It is conceivable that the stimulation parameters were not optimally matched to the individuals to elicit statistically and clinically relevant reductions of AVH. This is in accordance with the increasing support for the approach of personalized medicine in the treatment of schizophrenia. Examining individual differences could expose factors contributing to response. Accordingly, variability in brain structure, such as size, lateralization and gyrification might lead to unreliable estimations of the localization of the TPJ area. Although the International 10–20 EEG system we used for localization does take variability in head size into account, it is difficult to account for differences in brain morphology. Frameless stereotactic neuronavigation using anatomical MRI scans may allow for more accurate individual targeting. Few rTMS trials for the treatment of AVH applied this method of localization, with mixed results [25], [30], [31].

Not only brain structure, but also patterns of associated brain activation during AVH may differ between patients [61], [62]. One study that used fMRI-guided rTMS, based on individually defined areas of maximal activation during AVH, did not reveal additional benefits [28]. Homan et al. [63] on the other hand, assessed resting state perfusion in patients prior to treatment with rTMS. Those patients who eventually responded to rTMS treatment were characterized by increased cerebral blood flow in the left STG, suggesting that resting-state activity of STG could serve as a bio-marker of treatment response in schizophrenia patients with AVH. In a PET study by Klírová et al. [29], resting regional brain metabolism has been used for stereotactic neuronavigation to determine the optimum area for stimulation, and found this method to be superior over the traditional method and sham stimulation. Albeit it requires further clarification, assessment of functional brain activity before treatment may be valuable in the prediction of treatment success.

Functional neuroimaging studies can also provide information about the effect of rTMS on the underlying neural basis of AVH. However, only few studies have assessed brain activation before and after treatment. In a subgroup of present study sample, resting state connectivity was investigated. Reduction in hallucinations in the active rTMS group was correlated with an increased connectivity between the left TPJ area and the right insula [51]. Kindler et al. [36] assessed patients with AVH before and after treatment. The active treatment group showed both clinical improvement and decreased blood flow in the primary auditory cortex, left Broca’s area and the cingulate gyrus, whereas no changes in activity occurred in the sham group. So indeed, the left TPJ area appears an accurate target for influencing brain activity within the AVH network. One study that investigated the effect of rTMS on Broca’s area did not observe changes on a clinical or neural level [64]. This supports the idea that the speech perception areas in the temporal cortex, rather than speech production areas in the frontal cortex are important for the experience of AVH [65].

Since many of the rTMS studies in patients with AVH are characterized by small sample sizes, we recommend the initiation of large multi-center trials, and the validation of practical and reliable clinical measures in order to enhance power and draw stronger conclusions about the utility of rTMS in reducing AVH. To improve blinding, studies should employ sham coils that mimic the scalp sensation produced by real rTMS.

To summarize, although we observed a trend for AVH reduction in the left rTMS group as measured by clinicians, this improvement was not confirmed with self-reported hallucination scores. We thus conclude that we did not find evidence for the efficacy of left-sided rTMS, compared to sham stimulation. Moreover, bilateral rTMS was not superior over left-sided rTMS or sham in improving AVH. Mean self-reported hallucination scores, and negative and positive emotional content as measured with AHRS and PANAS were reduced during rTMS treatment and over the course of a three-month follow-up period, but its overall effect did not seem to be specific to either rTMS of the left or bilateral TPJ area, or sham treatment. Optimizing treatment parameters may yield more convincing evidence for efficacy of rTMS treatment of AVH. Moreover, for future studies it is recommended to incorporate evaluation of treatment effects with neuroimaging. And in order to develop individually tailored treatments, studies should focus on individual differences that may influence treatment response.

## Supporting Information

Checklist S1 **CONSORT checklist 2010.**
(PDF)

Protocol S1 **The approved protocol of the study.**
(PDF)

Data S1 **All data of the participants.**
(SAV)
