# Supplementary material for: Short and Long Term Effects of Left and Bilateral Repetitive Transcranial Magnetic Stimulation in Schizophrenia Patients with Auditory Verbal Hallucinations: A Randomized Controlled Trial
Source: PLoS One. 2014 Oct 20;9(10):e108828. doi: 10.1371/journal.pone.0108828 (PMC4203691; doi:10.1371/journal.pone.0108828)

METC
Reg.nr. Corsa
Mod. 57530
Datum ontvangst
11-3-2006
Casenr.
06.052
Dossiernr.
METc06.052

Mechanism and efficacy of low frequency rTMS treatment in schizophrenic patients with auditory hallucinations: An fMRI study

A. Vercammen, MSc (Researcher, BCN NeuroImaging Center, UMC Groningen)

L.M. Hoekert, MSc (Researcher, BCN NeuroImaging Center, UMC Groningen)

J.A. Jenner, MD, PhD (Psychiatrist, Department of Psychiatry, UMC Groningen)

R.E. Hoffman, MD (Professor, Department of Psychiatry, Yale University Medical School)

J.A. Den Boer, MD, PhD (Professor, Department of Psychiatry, UMC Groningen)

C.J. Slooff, MD, PhD (Psychiatrist, GGZ Drenthe and Department of Psychiatry, UMC Groningen)

H.C. Klein, MD, PhD (Psychiatrist, GGZ Groningen and Department of Nuclear Medicine and Molecular Imaging, UMC Groningen)

R. Knechtering, MD, PhD (Psychiatrist, Department of Psychiatry, UMC Groningen)

R. Bruggeman, MD, PhD (Psychiatrist, Department of Psychiatry, UMC Groningen)

A. Aleman, PhD (Professor, BCN NeuroImaging Center, UMC Groningen)

Protocol ID

METc 2006/052

Project Title

Mechanism and efficacy of low frequency rTMS treatment in schizophrenic patients with auditory hallucinations: An fMRI study

Coordinating investigator/project leader

Dr. A. Aleman

BCN NeuroImaging Center, A. Deusinglaan 2, 9713AW Groningen

Tel. 050-3638798

e-mail: a.aleman@med.umcg.nl

Principal investigator(s)

Ans Vercammen

BCN NeuroImaging Center, A. Deusinglaan 2, 9713AW Groningen

Tel. 050-3638999

e-mail: a.vercammen@med.umcg.nl

Independent physician(s)

Dr. B. M. de Jong

Tel. 050-3612644

e-mail: b.m.de.jong@neuro.umcg.nl

TABLE OF CONTENTS

A. INTRODUCTION 5
1. Schizophrenia and auditory hallucinations 5
2. rTMS treatment of auditory hallucinations 6
B. GENERAL RATIONALE AND RESEARCH GOALS 8
1. rTMS treatment effects on cerebral hemodynamics and behavior 8
2. Language and emotion lateralisation in schizophrenia 10
3. Hypotheses 11
C. SUBJECTS 13
1. Number of participants 13
a. Study in healthy subjects 13
b. rTMS treatment study 14
2. In-and exclusion criteria 15
a. Study in healthy subjects 15
b. *rTMS treatment study* 15
D. METHODS 17
1. Transcranial magnetic stimulation (TMS) 17
a. TMS mechanism 17
b. Repetitive TMS 18
c. TMS equipment 18
d. Ethical and safety issues 18
2. Magnetic resonance imaging (MRI) 19
a. Anatomical scan 19
b. Functional MRI 19
c. FMRI equipment 20
d. Data-analysis 20
e. Ethical and safety issues 20
E. EXPERIMENTAL PROCEDURES 21
1. Study in healthy subjects 21
2. rTMS treatment study 22
F. TESTS AND TASKS USED IN THE PROTOCOL 26
1. Psychopathology and hallucination rating instruments 26
a. Directed psychiatric interview: PANSS 26

b. Hallucination change score 26
c. Auditory hallucination rating scale 27
d. Beliefs about voices questionnaire 27
e. Positive and negative affect scale of hallucinations 28
2. Language and emotion lateralisation tasks 28
a. Prosodic discrimination of emotion task 28
b. Emotional facial perception task 29
c. Syllable counting task 30
3. Auditory verbal processing tasks 30
a. Metrical stress evaluation task 30
b. Perception-imagery interaction task 31
4. Resting state fMRI 33
G. POWER CALCULATION 34
H STATISTICAL ANALYSIS 34
H. GENERAL ETHICAL CONSIDERATIONS 35
1. Regulation statement 35
2. Recruitment and consent 35
3. Benefits and risks assessment 36
4. Compensation for injury 37
5. Incentives 37
I. ADMINISTRATIVE ASPECTS AND PUBLICATION 38
J. REFERENCES 39
K. APPENDICES 45
1. Appendix 1: Information letter for patients 45
2. Appendix 2: Information letter for participants 49
3. Appendix 3: Informed consent form 52
4. Appendix 4: Insurance information 53
5. Appendix 5: Flow chart 54
6. Appendix 6: BAVQ 55
7. Appendix 7: PANAS-hallucinations 57
8. Appendix 8: AHRS 60
9. Appendix 9: Recruteringsposter 62

A. INTRODUCTION

1. Schizophrenia and Auditory Hallucinations

Schizophrenia is a grave mental illness that ranks very high among causes of long term disability. As this disease usually manifests itself between the ages of 16 and 30 years, it affects individuals at an often pivotal time in their personal, professional and social development. Symptoms of this illness are traditionally categorized in three major types, namely cognitive impairment (e.g. deficits in attention, learning and memory, executive functions), negative symptoms (diminishing or loss of function in the behavioral and/or emotional domain including anhedonia, alogia and apathy) and positive symptoms (e.g. bizarre behavior, false beliefs, perceptual experiences not shared by others). Among the latter category, auditory hallucinations are a relatively prominent occurrence. They refer to a sensory experience during an awake state, which occurs in the absence of corresponding external stimulation of the relevant sensory organ, that resembles a veridical percept, and is not amenable to voluntary control by the subject. About 50 to 70% of all patients with schizophrenia experience auditory hallucinations, often in the form of speech or 'voices' (Andreasen & Flaum, 1991).

There has been considerable interest in relating this phenomenon to underlying brain structures and functions. Neuroimaging methods have enabled the identification of brain areas specifically involved in auditory hallucinations. Regions repeatedly implicated include the bilateral superior temporal cortex (STG) and medial temporal

cortex (MTG), left (para)hippocampal areas, right middle and inferior frontal areas and the anterior cingulate. Although rather controversial, auditory hallucinations have been associated with primary auditory cortex activation. Dierks et al. (1999) found primary cortex activation of only the dominant hemisphere during auditory hallucinations, but van de Ven at al. (2005) observed this activation bilaterally. However, hallucinations are not merely fostered by cortical regions. Activations in subcortical areas such as the (ventral) striatum and limbic regions have also been observed (Shergill et al., 2005; van de Ven et al., 2005; Copolov et al., 2002; Woodruff et al., 1997; Silbersweig et al., 1995). Over recent time, evidence has been accumulating of a substantial thalamic involvement in the disorder as well (Yasuno et al., 2004; Talvik et al., 2003). Neuropathological and in vivo imaging studies in schizophrenia have identified several structural and metabolic abnormalities in the thalamus. The thalamus has been implicated in top-down auditory attention (Frith & Friston, 1996), which implies that thalamic pathology may contribute to a deficit in sensory processing and be related to psychotic symptomatology.

2. rTMS Treatment of Auditory Hallucinations

Transcranial magnetic stimulation (TMS) is a non-invasive technique that enables safe, relatively painless focal brain stimulation in humans. A sufficiently strong and short pulse of current passing through a coil placed over a person's head generates rapidly changing magnetic pulses, which penetrate the skull and reach the brain fairly unattenuated. These pulses induce a secondary ionic current in the brain. In this way, TMS influences intracortical excitability. In repetitive TMS (rTMS) a train of pulses of

the same intensity is delivered to a single brain area at a given frequency of 1 Hz or higher. The effect it procures depends on the exact frequency. Low frequencies can suppress excitability of cortical neurons. This observation suggests a therapeutic value against pathological neuronal hyperactivity. Accordingly, symptom reductions following rTMS application have been observed in several neurological and psychiatric disorders, such as depression and bipolar disorder. Moreover, a number of studies on the application of low frequency rTMS over the left temporo-parietal cortex of patients suffering from auditory hallucinations have reported positive results. A robust amelioration of symptoms was found, lasting for several weeks following treatment cessation (Poulet et al., 2005; Chibbaro et al., 2005; Hoffman et al., 2005; Hoffman et al., 2003; Lee et al., 2004; d'Alfonso et al., 2002)[1]. Nevertheless, some experimental trials have yielded null effects or mixed results (Schönfeldt-Lecuona et al., 2004; McIntosh et al., 2004). On closer inspection, methodological variables may explain the absence of effect in these studies (e.g. intermittent rather than continuous stimulation). Taken together, results have generally been positive. Indeed, a recently conducted meta-analysis of 9 sham-controlled treatment studies has provided support for the efficacy of the treatment in reducing hallucination severity ratings. A mean effect size of .82 was observed (Aleman, Sommer & Kahn, submitted). However, the exact underlying mechanism of the observed improvement remains elusive as yet. TMS modulates intracortical excitability and influences distant cortical and subcortical structures along specific connections. Several studies in humans, combining rTMS with functional neuroimaging techniques have detected suppressed blood flow after slow (1 Hz) rTMS, and increased blood flow after

[1] A member of the present investigative team (Dr. A. Aleman) was co-investigator in the latter study, conducted at the University Hospital, Utrecht.

rapid (10-20 Hz) rTMS in the stimulated area. It is thus conceivable that rTMS decreases pathologically enhanced activity in certain brain regions connected to the stimulated superior temporal gyrus (STG), as well as activity in the STG itself.

B. GENERAL RATIONALE AND RESEARCH GOALS

1. rTMS Treatment Effects on Cerebral Hemodynamics and Behavior

Taking into account the debilitating character of the illness and the encumbering effect of auditory hallucinations on everyday life, research on a possible treatment mechanism and efficacy is surely legitimate. The objective of the current investigation is to map the hemodynamic changes in specific brain regions after rTMS treatment compared to the baseline measurement before the treatment. Pre- and post-treatment fMRI scans will be performed in order to asses the hemodynamic changes associated with symptom amelioration in patients with medication resistant hallucinations. Changes in the experience of symptoms will be assessed with a number of rating scales pertaining a.o. to frequency, severity, salience & emotional impact of the hallucinations. These scales and the auditory processing tasks used in this paradigm are described in a later section.

All but one of the behavioral treatment studies of rTMS effects on hallucination symptoms have restricted stimulation to the left temporal area. The effects of such treatments appear to be largely restricted to a decrease in hallucination frequency.

However, there is evidence of bilateral temporal cortex involvement in the genesis of auditory hallucinations (see above). Left superior temporal areas are hypothesized to be involved in 'speech' perception during hallucinations, i.e. the comprehension of the phonological and semantic characteristics of the hallucinated content. Right temporal areas are more associated with the processing of prosody and the emotional response to the often derogatory or hostile form of the hallucinations. Therefore, in the current experiment we wish to investigate whether a combination of both left and right temporal stimulation has more beneficial effects than simple left sided stimulation for the same length of time. It is possible that a combined treatment allows a more complete management of the symptoms, effectively targeting frequency, form and emotional salience.

Thus, patients will receive 1 Hz rTMS treatment over 6 days. One group will receive only left temporal stimulation, one will receive bilateral temporal stimulation and a third group will serve as a control group, receiving sham rTMS (i.e. a placebo). First, we will administer several hallucination rating scales once before and once after treatment, which will allow the assessment of the therapeutic efficiency of both treatment types compared to a placebo treatment on a behavioral level. Secondly, all patients will undergo an fMRI scan once before the start of the treatment and once when the treatment is completed. Pre and post treatment scans can thus be compared within groups and between groups. Our fMRI investigation thus comprises a first step towards a better understanding of the neurocognitive underpinnings of a treatment, for which evidence exists attesting its effectivity (unilateral left rTMS) and a rather exploratory endeavor towards a new treatment protocol (bilateral rTMS). Subjects will perform a number of

auditory processing tasks while being scanned. These include a metrical stress evaluation task, which probes the performance of the 'inner voice' and 'inner ear', and has been shown to activate auditory language processing areas (superior temporal gyrus and sulcus) in normal subjects (Aleman et al., 2005). All subjects will also perform an auditory perception/imagery interaction task. This task is sensitive to the balance between imagery and perception, which is hypothesized to be aberrant in hallucinating patients (Aleman, Böcker, Hijman, de Haan & Kahn, 2003). A resting condition will be added as well, allowing the recording of a resting-state fMRI scan. This procedure is used to obtain an image of functional networks of the unstimulated brain. For a description of the specific tasks and procedures used, see below.

2. Language and Emotion Lateralization in Schizophrenia

The use of both left sided and right sided rTMS treatment provides an excellent opportunity to test a number of hypotheses regarding the lateralization of emotional processing in schizophrenia. Particular disturbances have been hypothesized in schizophrenia with regard to the processing of emotional material (Pinkham, 2003; Lee, 2004). Among these are disturbances in recognition of facial affect and perception of affective prosody in patients with schizophrenia (Edwards, 2001; Leentjens et al., 1998). According to one study, this deficit is as pronounced as in neurological patients with right hemisphere lesions (Ross et al., 2001). In order to examine these disturbances we will include some cognitive tasks, carried out after six days of either left or bilateral stimulation. Subjects will be asked to perform an affective prosody task, in which they

are required to focus on either semantics or prosody and decide which emotion is expressed. An emotional facial expression task is also included. Neuroimaging studies showed that the superior temporal gyrus is associated with emotional prosody (Mitchell, 2003; Meyer, 2002) and with the perception of emotion from facial expression (Adolphs, 2002; Allison, 2000). We predict that the schizophrenia patients will show TMS effects on these tasks (increased reaction time and number of errors) after stimulation of the left hemisphere as compared to the placebo group, indicating a decreased lateralization of function. Whereas the right hemisphere may be more critical than the left in processing emotional prosody, there are reasons to assume that this neural substrate is bihemispheric. We predict that effects will be larger after bilateral stimulation. In order to further examine lateralization of language functions in schizophrenia, we will also include a syllable counting task, normally subserved by the left-hemisphere. We predict that performances on this task will be negatively influenced by TMS stimulation after left and even more after bilateral stimulation. In accordance with the right shift theory, earlier described in schizophrenia (Annett, 1998; Annett, 1999; Crow, 2000).

3. Hypotheses

To summarize we will outline the hypotheses to be tested in the present study. This investigation was designed to test one main research question and a number of related hypotheses. The primary aim concerns the comparison of the neural response on the auditory-verbal tasks in patients before and after rTMS treatment, in order to assess the expected normalization of higher order auditory processing in the brain. Specifically,

we expect a decrease of hyperactivity in the secondary auditory cortex as a result of the rTMS intervention. To obtain a more exhaustive interpretation of the hypothesis, we will also contrast the fMRI findings from patients with results from normal, non-psychiatric subjects. This is warranted, especially since some of the tasks have never been used in an fMRI setting before. However, we do have specific expectations regarding the patterns of activity present in normal subjects while performing the auditory processing tasks. Based on earlier research with similar tasks, we hypothesize that activation will arise in the supplementary motor area, inferior frontal gyrus and insula. We also expect strong temporal (superior temporal gyrus and sulcus) activation in the perceptual conditions and a smaller posterior superior temporal activation in the imagery conditions. In the resting state images, we expect to find distinct patterns of low-frequency coherences across the brain. These coherences are indicative of an "idling" mode of interactions between functionally integrated regions. De Luca, Beckman, Stefano, Matthews & Smith (2005) describe five such spatio-temporally distinct patterns: visual processing areas (striate cortex), internal monitoring and consciousness areas (precuneus, anterior pole, thalamus, hypothalamus and medial parietal lobe), motor control and somatosensory areas (post-central gyrus, insula and midline cingulate), action perception areas (dorsal parietal, occipital and prefrontal regions) and object perception areas (ventral parietal, occipital and prefrontal regions). Comparing task performance and resting state fMRI images may thus yield insight into functional networks in the brains of hallucinating schizophrenia patients and how they relate to normal subjects before and after rTMS treatment. Healthy subjects will be tested in an experiment run before the commencement of patient testing.

A number of additional and related hypotheses will be tested as well. We will investigate the efficacy of bilateral rTMS compared to unilateral left rTMS on a number of hallucinating rating scales (self-ratings by patients and a psychiatric interview). We expect additional clinical benefit from the bilateral stimulation, as it comprises a more comprehensive treatment and targets both analytical linguistic (left) regions as well as more emotional language processing (right) regions. A third hypothesis concerns the effect of rTMS on cognitive task performance. Specifically we will test the effect of rTMS treatment on tasks probing language and emotion processing.

A flow chart has been added to the appendix, which should clarify the different steps in the investigation and the different groups of subjects participating in each step.

C. SUBJECTS

1. Number of Participants

(a) Study in Healthy Subjects

We seek to include a sample of 16 healthy volunteers to participate in the fMRI pilot investigation, in order to establish the neural correlates of auditory perceptual expectations in healthy people. These subjects will be recruited locally, with the use of posters and/or pamphlets. Subjects will be matched to the patient population based on gender, age and education level.

(b) rTMS Treatment Study in Patients

All subjects will be recruited from three local psychiatric hospitals (GGZ Drenthe in Assen, GGZ Groningen in Winschoten, and the Department of Psychiatry at the UMCG). A total of 48 subjects will take part in the study, which is divided into an rTMS part and an fMRI part. It is known that a significant percentage of patients fail to complete fMRI measurements, due to varied reasons (e.g. a claustrophobic attack, inability to follow task instructions). Hence, we will take into account that a group of subjects, who are admissible for study inclusion, will not be able to complete the fMRI part of the study. Therefore, we will aim for an inclusion of 12 subjects per group in the fMRI part of the study, and allow 4 additional subjects in the rTMS part of the study alone.

rTMS part of the study. *For this part of the study, we seek to include 16 subjects in each group. One group of 16 patients will receive 6 days of left sided rTMS. Another group of 16 patients will receive 6 days of bilateral stimulation. A third group of 16 patients will constitute the placebo control group and receive sham stimulation. Of these subjects 12 subjects will also take part in the fMRI part of the study (see below).*

fMRI part of the study. *For this part of the study, we seek to include 12 subjects in each group. These subjects will complete fMRI measurements in addition to rTMS treatment.*

~~The treatment study will be conducted with a total sample of 36 patients, diagnosed with schizophrenia. They will be recruited from three local psychiatric~~

~~hospitals (GGZ Drenthe in Assen, GGZ Groningen in Winschoten, and the Department of Psychiatry at the UMCG). Three groups of patients will take part in the experiment. Patients will be allocated to the conditions on a random basis. One group of 12 patients will receive 6 days of left sided rTMS. Another group of 12 patients will receive 6 days of bilateral stimulation. A third group of 12 patients will constitute the placebo control group and receive sham stimulation. Patients in each of the groups will be matched on relevant characteristics such as gender, age, education, illness duration, illness severity and medication status. We take into account a general attrition rate of 20%, ensuring a sufficient number of participants to complete the full procedure.~~

2. In-and Exclusion Criteria

(a) Study in Healthy Subjects

Subjects should be free from neurological and psychiatric disorders. Further exclusion criteria consist of MRI contraindications such as intracerebral or pacemaker implants, inner ear prosthesis or other metal prosthetics/implants and claustrophobia. For safety reasons we will also exclude female participants who may be pregnant.

(b) rTMS Treatment Study in Patients

Inpatients as well as outpatients can participate. All patients should meet the diagnostic criteria for schizophrenia, schizophreniform or schizoaffective disorder, and should report fairly frequent auditory hallucinations (at least daily). This inclusion

information will be based on the patient's medical file and a structured psychiatric interview (Schedules for clinical Assessments in Neuropsychiatry, SCAN) conducted at intake). Patients should also meet the criteria for medication resistance, defined as persistent auditory hallucinations occurring in face of at least two adequate trials of antipsychotic medication. Patients stay on their regular medication for the complete duration of the study. Exclusion criteria include rTMS and MRI contraindications (e.g. a personal or family history of epileptic seizures, past neurosurgical procedures, intracerebral or pacemaker implants, inner ear prosthesis or other metal prosthetics/implants), neurological disorders, history of significant head trauma, severe behavioral disorders, claustrophobia and current substance abuse. For safety reasons we will also exclude female participants who may be pregnant. Subjects must show decision competence. Patients admitted under BOPZ regulation are not allowed to participate. Furthermore, the ability to give informed consent will be assessed by the patient's psychiatrist or clinical psychologist. Only subjects who are fully capable of making their own decision regarding participation in the research will be included. Patients with an active psychosis, i.c. who have lost contact with reality, cannot participate due to a lack of concentration and understanding necessary for completion of the procedure.

D. METHODS

1. Transcranial Magnetic Stimulation (TMS)

Here we provide a brief general description of the technique to be applied in the current study. Details of the specific parameters and the precise procedure are given in a further section (E. Experimental Procedures).

(a) TMS Mechanism

TMS is an innovative technique, which uses the principle of inductance to get electrical energy across the skull by means of a train of brief magnetic pulses. It involves placing a coil on the scalp and passing a powerful and rapidly changing current through it. When the amplitude of the magnetic field varies in the order of 200 microseconds, a second electrical current will be induced in nearby conductive tissue, which will excite neurons in the brain (Pascual-Leone, Walsh & Rothwell, 2000) . The relevant stimulation parameters in the present context consist of intensity and frequency. The intensity is typically given as a percentage of the threshold intensity for evoking movement of the thumb by stimulating the contralateral primary motor cortex (Wassermann, 1998) . For quantifying the individual cortical excitability, i.e. the motor threshold (MT), five out of ten consecutive single-pulse TMS should elicit an observable contraction of the muscle. Moreover, stimulation frequencies less than 5 Hz are argued to cause disruption in ongoing neural signaling, while higher frequency stimulations can have the opposite effects. Since the effect of single TMS is transient, the duration of the effect usually

extends only a few tens of milliseconds (Stewart, Ellison, Walsh & Cowey, 2001) . Extensive safety experiments have demonstrated that TMS is a safe method for investigating the functional organization of the brain (Wassermann, 1998; Pascual-Leone, 1993).

(b) Repetitive TMS

In repetitive TMS (rTMS) a train of pulses of the same intensity is applied to a single brain area at a given frequency that can range from one stimulus per second to 20 or more. This technique (at 1 Hz) is used in present project.

(c) TMS Equipment

We will use a MagStim Rapid magnetic stimulator (MagStim Co, Whitland , UK) with a figure of eight coil with a diameter of 70 mm for each loop.

(d) Ethical and Safety Issues

Extensive safety experiments have shown that TMS is a safe technique to investigate the functional organization of the human brain (e.g. Wasserman, 1996). The rTMS technique uses a magnetic field of 2 Tesla. Therefore subjects with metal intracerebral implants , cardiac lines, pacemakers etc. need to be excluded from research using TMS equipment. A history of severe cerebral trauma, epilepsy, a family history of epilepsy or the use of seizure-threshold lowering drugs are also exclusion criteria.

2. Magnetic Resonance Imaging (MRI)

MRI is a non-invasive, patient-friendly way to study and visualize the structure of the living human brain. This method measures the concentration of water in different tissue types, based on the magnetic resonance of protons (Andrews, 1992).

(a) Anatomical Scan

Every MRI investigation requires the production of an anatomical scan, which takes approximately 15 minutes. These scans result in high-resolution images with large contrasts between grey and white matter and cerebrospinal fluid. This allows the identification of separate brain regions and the determination of the subject's specific cerebral anatomy. These data are used to localize activation observed during functional imaging.

(b) Functional MRI

fMRI permits the visualization of brain regions activated during the performance of a specific (mental) act in perception, cognition and motor control. The method is based on a change in local blood oxygen perfusion in the activated region. The measure of interest is called blood oxygenation level dependent (BOLD) signal. Due to a poor signal to noise ratio, a single measurement is insufficient. Thus, during a single fMRI scan, the brain is actually scanned a large number of times in order to improve the signal-to-noise ratio.

(c) The (f)MRI equipment

All fMRI experiments will be run with the use of a 3 Tesla MR system (Philips Intera) at the BCN NeuroImaging Center, Groningen.

(d) Data-analysis

fMRI data are analyzed by way of a statistical software package (Brainvoyager, AFNI; http://afni.nimh.nih.gov/afni/). Every analysis contains a number of consecutive steps. First the data are corrected for movement artifacts. Then the data are examined for changes in BOLD signal compared to a previously determined baseline. These changes are a measure for brain activity attributable to specific task performance. To determine the anatomical position of activated brain regions fMRI images are superimposed on the anatomical images.

(e) Ethical and Safety Issues

- Subjects are exposed to a high static magnetic field of 3 Tesla. Ample experience with such field strengths yield no indication of adverse effects on humans.
- The performance of an anatomical scan may lead to the detection of a clinically relevant cerebral anomaly. In the case of such a finding, the data will be evaluated by a trained neuroradiologist. When a relevant medical malformation is found, the subject will be informed through his/her general practitioner. Subjects will only be included in the research if they accept to be informed of any serendipitous

pathological findings. A statement to that effect must be signed before entrance in the study.

- Patients with metal implants or prostheses will be excluded from the study.
- Female participants are required to sign a statement indicating that they are not pregnant (to their knowledge). Although no adverse effects on the unborn foetus have been described, pregnant women will be excluded from the study, for safety reasons.
- Any indication of claustrophobic or epileptic activity is a cause for immediate cessation of the experiment and further exclusion from the research.
- In order to protect the subjects' hearing, protective headphones will be provided.

E. EXPERIMENTAL PROCEDURES

1. Study in Healthy subjects

The goal of this experiment is to apply the auditory processing tasks in an fMRI setting in order to assess normal processing in the non-diseased brain. This will allow for a comparison with behavioral and hemodynamic variables obtained from patients before the start of rTMS treatment for hallucinations and after this treatment.

Subjects will each be scanned while performing a number of auditory cognitive tasks and while at rest. The auditory processing tasks will performed by this group to provide a baseline measurement both behaviorally and hemodynamically to compare the

patients with before and after they receive treatment. The specific tasks are described in more detail in another section (see below). Importantly, to overcome the disadvantages of the acoustic scanner noise generated by rapid gradient switching in echo planar imaging (EPI) in auditory fMRI experiments, we will apply a sparse temporal sampling technique. The acoustic noise generated by a typical high-field MRI scanner is often sufficiently loud (in excess of 120 dB SPL) to make it difficult for subjects to hear auditory stimuli that might otherwise be expected to produce changes in BOLD activation. The technique we will apply is widely used to overcome the influence of scanner noise on stimulus presentation during fMRI, as it temporally separates EPI scanner noise from the experimental sounds, taking advantage of the fact that the peak of the hemodynamic response lags the stimulus by several seconds (Edmister et al., 1999, Hall et al., 1999 and Moelker and Pattynama, 2003). Data acquisition follows a silent interval during which experimental sounds are presented. Sequence timing is chosen such that the peak of the stimulus-related BOLD response coincides with data acquisition.

The total session time in the scanner approximates 50 minutes.

2. rTMS Treatment Study

Before the start of the trial (i.e. no earlier than 5 days before the start of the rTMS treatment) all patients are assessed with a number of questionnaires and a psychiatric interview, pertaining to experience of hallucinations (severity, frequency, emotional salience, etc). These include the PANSS, HCS, AHRS, BAVQ and (adapted) PANAS and are covered in more detail in a later section of this protocol. The same instruments

will be used for assessment on the last day of treatment. Before the start of the first rTMS session an fMRI scan will be obtained from all patients. During scanning the patient will perform the auditory processing tasks. For all fMRI scans, a sparse sampling technique will be used, as described in a previous section. A resting state fMRI scan will also be obtained. Subsequently, the rTMS treatment will be performed over a period of 6 days, with two stimulation sessions per day (once in the morning, once in the afternoon). On days that the rTMS treatment is performed outpatients (including those in the placebo group) will be admitted to the day-clinic or will be admitted to the inpatient ward on a voluntary basis, according to their own wishes and convenience (e.g. patients not living in the vicinity of Groningen may prefer to stay overnight for the duration of the investigation, whereas patients recruited locally may want to utilize the outpatient facilities and return home each evening). Participants, who were already inpatients, receive their regular care. Patients receive treatment at either one of the three facilities (UMC Groningen, GGZ Assen or GGZ Winschoten). As two sessions are required per treatment day, it would be beneficial for the patient to return to the ward, where he/she can relax and enjoy the amenities (e.g lunch will be provided) until the afternoon session. One group of patients will receive 6 days of left temporal stimulation (two series of 3 consecutive days, separated by a weekend hiatus). During each session the patient will be stimulated at a frequency of 1 Hz, for the full duration of 20 minutes. The second group will receive 6 days of bilateral stimulation, i.e. simultaneously to the left and right temporal lobes (in two series of 3 consecutive days). Session duration is set to 10 minutes, rather than 20, in order to equalize the number of pulses each patient receives on a daily basis. This also ensures that the total treatment duration will be the same in both

groups. Parameters for the rTMS treatment conform to safety guidelines used by previous studies. Stimulation will be carried out with a MagStim Rapid stimulator system, with a 70 mm figure-eight coil. Stimulation intensity will be set at 90% of the motor threshold (cf. Hoffman, 2005; Brunelin, 2005). Motor threshold will be identified as the minimum magnetic field strength required to produce left thenar muscle activation by single transcranial magnetic pulses delivered to the motor cortex for at least 5 of 10 trials. The location of the stimulation will be determined with the help of a neuronavigator, based on the functional MRI scan on which the specific speech perception areas of the temporal lobe can be identified individually for each subject. Coil positioning will be achieved using Nena software (Eggers et al., 2004). Stimulation of the right temporal lobe will occur on the homologue area of the right hemisphere. A placebo control group of patients will be included. This group will receive 'sham' stimulation to the left temporal lobe. In this case, the stimulation will conform to the same parameters (equal number of sessions and session duration etc.) as in the real TMS condition, but a sham coil will be used. This coil is visually indistinguishable from the real coil and produces the same characteristic clicking sound, yet induces no magnetic field and does not influence the underlying cortex. All rTMS treatment sessions will be conducted with a physician on call and available, should intervention be required.

The TMS administrator will be aware of the treatment condition each patient belongs to. Based on assessment of the likelihood that the administrator will notice whether she is using the real or sham coil, it was decided that she should not be blind to the treatment condition of participants. Because of this, the TMS administrator will not be involved in the allocation of patients to the conditions, nor in the assessment of the

hallucination scales before and after treatment. A different investigator will thus allocate patients to the respective conditions on a random basis. Patients are not aware of the condition they are in (real or sham). A third investigator will be in charge of assessment of the hallucination rating scales before and after the treatment with TMS.

When the treatment has been completed, each subject will undergo a second fMRI scan during which the same cognitive tasks will be performed. This scan will be contrasted with the baseline scan obtained pre-treatment. Care will be taken to ensure that the scan is scheduled no later than two days after termination of the treatment. The questionnaires and interview conducted at the beginning (PANSS, AHRS, HCS, BAVQ and adapted PANAS) will be re-assessed on the last day of treatment, to gauge changes in hallucination ratings. Additionally, subjects will be questioned on the experience of the treatment, namely whether they were aware of the condition they were allocated to, whether they felt the treatment influenced their brain activity and whether they observed any muscle contraction during the treatment.

In order to assess the long-term effects of the treatment, on the level of the subjective experience, semi-regular check-ups will occur after completion of the treatment. During such an evaluative session the patient will fill out the aforementioned questionnaires (AHRS, BAVQ, PANAS, HCS), to check (re-)occurrence of symptoms. In an informal conversation, the patient will further be given the opportunity to comment on his/her subjective experience regarding auditory hallucinations in the recent past and on any possible side effects of the treatment. Four such sessions will be scheduled: one week, four weeks, three months and six months after completion of the treatment.

Patients will not be made aware of the condition they were in (sham or real TMS) until after the last evaluation session.

F. TESTS AND TASKS USED IN THE PROTOCOL

1. Outside the Scanner: Psychopathology and Hallucination Rating Instruments

(a) Directed Psychiatric Interview: PANSS

Positive and negative syndrome scale. This semi-directive interview was developed and standardized for the typological and dimensional characterization of schizophrenia. It consists of 30 items, each scored on a 7-point scale and divided between a scale for positive symptoms, a scale for negative symptoms and a general psychopathology scale. The interview refers to a specific time period, usually the previous week. Information is collected from the patients themselves as well as primary care-givers and family members. Specific items probe hallucinatory experiences. A hallucination score can thus be obtained, indicating the severity and frequency of hallucinations.

(b) Hallucination Change Score (HCS)

The patient is asked to generate a narrative description of his/her auditory hallucinations. This description refers to the 24 hours preceding the assessment and is scored as a 10. The HCS is then scored again after the treatment by requesting the patient

to construct a new narrative, again describing their hallucinations in the preceding 24 hours. This follow-up assessment then ranges from a score of 0, corresponding to no hallucinations to 20, corresponding to hallucinations twice as severe as the baseline. This measure was previously used by Hoffman et al. (2005).

(c) Auditory Hallucination Rating Scale (AHRS)

This scale was developed by Hoffman et al. (2003) and is used to assess hallucination frequency, number of distinct speaking voices, perceived loudness, vividness, attentional salience (the degree to which the hallucinations capture the attention of the patient), length of hallucinations (single words, phrases, sentences or extended discourse) and degree of distress. This scale, as well as the HCS provide a more detailed and fine-grained analysis of hallucination severity compared to the more general PANSS.

(d) Beliefs about Voices Questionnaire

The BAVQ is derived from the Chadwick & Birchwood (1994) cognitive model of the maintenance of auditory hallucinations. It measures beliefs, feelings and behavior associated with auditory hallucinations. It consists of 30 yes/no questions to be filled out by the patient (e.g. 'my voice wants to harm me', 'when I hear my voice, usually I am reluctant to obey it').

(e) Positive and Negative Affect Scale of Hallucinations

The specific scale used in this experiment is essentially identical to the PANAS. The PANAS is designed to measure Positive and Negative Affect. These are defined as two independent dimensions. Positive Affect (PA) reflects the extent to which a person feels enthusiastic, active, and alert. High PA is a state of high energy, full concentration, and pleasurable engagement. Low PA is characterized by sadness and lethargy. Negative Affect (NA) is a general dimension of subjective distress and unpleasurable engagement that subsumes a wide variety of aversive mood states. High NA is associated with anger, contempt, disgust, guilt, fear, and nervousness. Low NA is characterized as by a state of calmness and serenity. Ten items are used to measure each dimension. The scale comprises 20 items in total (10 measuring each dimension), scored on a 5 point Likert scale. Here, the instructions were adjusted to probe the emotional state during the perception of hallucinations, rather than during the current state. This scale will be administered to assess changes in the emotional experience of hallucinations as a result of treatment.

2. Outside the Scanner: Language and Emotion Lateralization Tasks

(a) Prosodic Discrimination of Emotion Task

The prosodic discrimination of emotion task has been validated and described by Vingerhoets et al. (2003), who demonstrated, using transcranial Doppler ultrasonography, that attention to emotional semantics activated the left hemisphere whereas attention to emotional prosody also strongly activated the right hemisphere in healthy subjects. The

task consists of sentences of neutral content pronounced in emotional tones of voice by two professional actors, a male and a female voice, to control for individual and/or gender differences in affective prosody (we have enough stimuli to make multiple parallel versions of the tasks). The digitized stimuli are of approximately equal length and presented by a computer at a rate of one sentence per 20 seconds through two computer sound boxes. During listening, the emotions to be discriminated (fear, anger and neutral) will be presented on the computer screen. In the prosody condition, subjects have to attend to the affective tone of voice and ignore the neutral semantic content. In the semantics condition, subjects have to attend to the affective semantic content and ignore the neutral tone of voice. As soon as they identified the emotion expressed in the sentence, either based on content or tone of voice, subjects are required to use the index finger of their right hand to make a "fear" response on a keypad or the middle finger to make an "anger" response or to press the spacebar for a "neutral" response. A computer records reaction times and number of correct answers.

(b) Emotional Face Perception Task

For the emotional face perception task we will use photographs of different individuals with either a fearful or an anger emotion expression or a neutral face, selected from two distinct face sets, namely the Ekman series, (Ekman & Friesen, 1976) and the Karolinska Directed Emotional Faces set, (Lundqvist et al., 1998). The faces are presented on a computer screen. Subsequently subjects are instructed to decide which emotion is expressed, by pressing one of three keys on a keypad. The computer records accuracy and reactions times.

(c) Syllable Counting Task

This task has been used in a number of brain imaging studies on covert language and has been found to significantly activate left hemisphere frontal regions (Poldrack et al., 1999; Price et al., 1997). During this task subjects are asked to count the number of syllables in words presented on a computer monitor. These stimuli consist of equal numbers of three-, four- and five-syllable words and are pseudorandomly presented. The words are chosen in order to avoid a possible visual recognition and correspond to familiar objects. Reaction times and number of correct answers are recorded by a computer.

3. Inside the Scanner: Auditory Verbal Processing Tasks

(a) Metrical Stress Evaluation

This tasks consists of 2 conditions, perception versus imagery. Subjects are required to indicate, for bisyllabic words, the syllable that carries the metrical stress. Dutch is a so-called stress-timed language, i.e. they distinguish between weak and strong syllables. For every word presented, subjects have to press the appropriate response button to indicate whether the metrical stress is on the first or the second syllable. In the perceptual version of the task, words are presented aurally via headphones and subjects can thus greatly rely on the externally provided stimulus. In contrast, in the imagery case, words are visually presented and subjects are required to imagine hearing the stimulus in

order to solve the task. The stress pattern has to be internally generated through the retrieval and activation of the phonological form. Obviously this condition has a stronger production component and can only be solved by listening to the 'inner voice' via the inner ear'. There is some indication that patients who suffer from hallucinations show (a) a central auditory processing deficit and (b) abnormalities of 'inner speech' (Rossell & Boundy, 2005; McKay, Headlam & Copolov, 2000; McGuire et al., 1996). Both processes can be evaluated with this task. Each condition has 60 trials. The duration of the task is estimated at 15 minutes.

(b) Perception-Imagery Interaction

The task is designed to assess the interaction between top-down processes (imagery) and bottom up perception, on the level of word processing. A single trial begins with the aural presentation of a spoken word, through headphones. The items consist of adjectives applicable to human beings. Three types of adjectives will be used, namely those with negative/derogatory, positive/appreciative or neutral meaning. Examples could be 'kind' (positive/appreciative), 'stupid' (negative/derogatory) or 'ready' (neutral) [2]. After hearing the word, a delay period of 2 seconds follows, wherein subjects are asked to 'image' the word in an auditory fashion, i.e. create an auditory image of the word in their mind. The last phase of the trial consists of a burst of white noise. On half of the trials no sound is added to the white noise. On the other trials a target word is superimposed on the noise, at the individual's predetermined auditory

---

[2] The emotion valence of the stimuli will be tested in an unrelated sample of individuals, using a questionnaire form with a Liker type rating scale. Participants will be asked to denote to emotion valence on a scale from -4 to +4, indicating respectively, extremely negative and extremely positive. Only items with a very high score will be selected for the positive stimulus group and items with a very low score will be considered for the negative stimulus group. Items scoring around zero will be considered neutral.

threshold. On half of these trials the presented word will be identical to the previously presented 'imaged' one. On the other trials the word will be a different one. Subjects are asked to press the appropriate response button to indicate whether or not they heard a word, and subsequently to vocally identify the word. A total of 180 trials will be used and the total duration of the task is estimated at 25 minutes, including the individual determination of the perceptual threshold. This procedure will yield several different response measures (reaction times, number of false positives, hits, misses and correct omissions, and some qualitative information, e.g. the kind of response on a false positive). The difference between the number of stimuli detected when the image was identical to the target and when it was not, is a measure for the interaction between imagery and perception (Aleman et al, 2003). If it is the case that hallucinatory experience is related to a distorted balance between imagery and perception, i.e. when imagery influences perception to a greater extent, this difference should be larger in individuals who suffer from hallucinations. We further take into account the emotional salience of the stimuli since it has been shown that the occurrence of hallucinations is linked to emotional events or stressors and that their content is affective, often derogatory in tone. Johns et al. (2001) for instance showed that hallucinating patients made more misattribution errors in a monitoring task of their own speech when the words spoken were derogatory. Patients with positive symptoms also show more interference for threat-related words on an emotional Stroop task than do patients without active psychotic symptoms (Epstein, Stern & Silbersweig, 1999). Normal individuals at risk for psychosis, i.e. those showing positive schizotypy also appear to suffer more interference on an emotional Stroop task. These findings point to increased affective reactivity of cognitive

disturbance in individuals with positive symptoms. We would thus expect the effect to be enhanced in the case of negative items, compared to positive or neutral ones in patients with schizophrenia.

4. Inside the Scanner: Resting State fMRI

This method relies on the capturing of fMRI images during a resting state, i.e. while no specific task is performed. Subjects are simply required to lie in the scanner with their eyes closed and relax without falling asleep. The procedure takes approximately 10 minutes. Studies have suggested that low-frequency fluctuations in resting fMRI data collected using BOLD contrast correspond to functionally relevant 'resting state networks' (RSNs). Analysis of these images thus allows the identification of varying patterns of signal coherence across the brain at rest (De Luca, Beckmann, De Stefano, Matthews & Smith, 2005). The association between the located activations and the unstimulated brain suggests a "default" or "idling" state of these functional networks. They potentially provide information on functional systems and their interactions. They also may prove useful to probe functional alterations in the brain as a consequence of disease. Liu et al. (2006) and Liang et al. (2006) for instance have observed a decrease in functional connectivity and decreases in regional homogeneity in schizophrenia patients during resting state. Resting state networks may also differ between hallucinating patients (before treatment) and patients who no longer or to a lesser degree experience hallucinations. We would expect a hyperactivity in networks including temporal auditory processing areas in patients frequently suffering from hallucinations compared to patients

who do not (or no longer) experience auditory hallucinations and compared to healthy persons.

The total duration of the session in the scanner approximates 50 minutes.

G. POWER CALCULATION

Previous research using a similar design and rTMS parameters (Brunelin et al., 2005) found significant effects of the treatment on behavioral variables (auditory hallucination rating scales). An effect size of 1.22 was observed. With the inclusion of 12 subjects in each condition a power of > .80 would be observed.

H. STATISTICAL ANALYSIS

To test the first hypothesis we will conduct a brainvoyager analysis to identify and compare regions activated by the performance of the auditory-verbal tasks. The efficacy of the rTMS treatment will be analyzed by means of an ANOVA on rating scale scores with 'group' as a between subjects factor with three levels (unilateral, bilateral or placebo stimulation). *Correlation analyses will be conducted between clinical variables obtained from the SCAN interview and (the first) PANSS interview and the observed treatment efficacy. Clinical variables consist of e.g. illness duration, number of hospitalizations, medication dose, other concurrent symptoms, general psychopathology.* The effect or rTMS on cognitive task performance will be analyzed by means of an

ANOVA on reaction times and accuracy with 'group' again as a between subjects factor with three levels.

I. GENERAL ETHICAL CONSIDERATIONS

1. Regulation Statement

The study will be conducted according to the principles of the Declaration of Helsinki (version 2004) and in accordance with the Medical Research Involving Human Subjects Act (WMO).

2. Recruitment and Consent

Healthy subjects will be recruited by way of posters and pamphlets distributed at the University Medical Centre and by word of mouth.

The psychiatrists of the participating hospitals will ask patients whether they would like to be approached by the investigators of the current research project. Patients who agree are then informed by the researchers (A.V., L.M.H and A.A.) of the precise goals and procedures of the experiment, by way of an information letter. Patients who show interest in participating are asked to contact one of the researchers. Contact information is given in the patient information letter. Before the start of the investigation informed consent will be obtained. Competence to give informed consent will be

assessed by the patient's psychiatrist or the assisting clinical psychologist. Participants are free to contact the project leader, independent physician or any of the researchers should they have further questions. Each subject will be allowed a week to consider their decision, but an extension is granted at request. The participant information letter and informed consent form are attached as appendices to this document. Patients are informed (see information letter) that the study consists of a blind trial, i.e. that they will be allocated by chance to one of the active conditions (with real rTMS) or the inactive condition (with sham rTMS). Upon completion of the procedure the actual condition the subject was allocated to, will be disclosed. For subjects randomized to the sham condition a subsequent nonblind active rTMS trial will be offered. It will be made clear that the treatment is in an experimental phase and has not been officially recognized, and that no guarantees can be given regarding clinical improvement.

3. Benefits and Risks Assessment

Patients can potentially derive benefit from partaking in the study, as beneficial effects are expected from rTMS treatment on the occurrence of auditory hallucinations. The risks involved are minimal. Extensive safety experiments have shown rTMS to be a safe technique, given that one conforms to safety guidelines outlined in e.g. Wasserman (1996). Serious complications such as seizures have only been documented in patients with a (family) history of epilepsy. There is also no evidence of adverse long term effects of MRI investigation. All subjects are required to fill out a safety form before entering the

investigation, which probes risk factors for MRI and TMS, such as implants or a history of epileptic seizures. The necessary precautions will be taken regarding the inclusion of subjects. Individuals subjected to significant risk are excluded from participation.

4. Compensation for Injury

The sponsor has a liability insurance which is in accordance with the legal requirements in the Netherlands (Article 7 WMO and the Measure regarding Compulsory Insurance for Clinical Research in Humans of 23rd June 2003) This insurance provides cover for damage to research subjects through injury or death caused by the study. See appendix 3 for further insurance information.

5. Incentives

Subjects receive no monetary or other reward for participating in the research, as they receive free treatment. However, a compensation for travel costs will be made available. Complying with remuneration standards, volunteers participating in the pilot investigation receive € 20.

I. ADMINISTRATIVE ASPECTS AND PUBLICATION

All data received from participants will be processed in a strictly confidential fashion. Researchers other than those immediately involved in data-collection only have access to fully anonymous files, which cannot be retraced to a specific individual. Data used for publication are also completely anonymous. All handling of personal data will comply with the Dutch Personal Data Protection Act (Wet Bescherming Persoonsgegevens).

G. REFERENCES

Adolphs R. (2002). Neural systems for recognizing emotion. Current Opinion in Neurobiology, 12, 169-177.

Aleman A., Formisano E., Koppenhagen H., Hagoort P., de Haan E.H.F., Kahn R.S. (2005). The functional neuroanatomy of metrical stress evaluation of perceived and imagined spoken words. Cerebral Cortex, 15, 221-228.

Allison T. (2000). Social perception from visual cues: role of the STS region. Trends in Cognitive Sciences, 4, 267-278.

Andreasen, M.C. & Flaum, M. (1991). Schizophrenia - The characteristic symptoms. Schizophrenia Bulletin, 17, 27-49.

Andrew, E.R. (1992). Nuclear magnetic resonance and the brain. Brain Topography, 5, 129-133.

Annett M. (1998). Handedness and Cerebral Dominance: The Right Shift Theory. Journal of Neuropsychiatry, 10, 459-469.

Annett M. (1999). The theory of an agnosic right shift gene in schizophrenia and autism. Schizophrenia Research, 39, 177-182.

Brunelin, J., Poulet, E., Bediou, B., Kallel, L., Dalery, J., D'Amato, Saoud, M. (in press). Low frequency repetitive transcranial magnetic stimulation improves source monitoring deficit in hallucinating patients with schizophrenia. Schizophrenia Research.

Chibbaro, G., Daniele, M., Alagona, G., Di Pasquale, C., Cannavò, M., Rapisarda, V., Bella, R. & Pennisi, G. (2005). Repetitive transcranial magnetic stimulation in schizophrenic patients reporting auditory hallucinations. Neuroscience Letters, 383, 54-57.

Copolov, D.L., Seal, M.L., Maruff, P. Ulusoy, R., Wong, M.T., Tochon-Danguy, H.J.& Egan G.F. (2003). Cortical activation associated with the experience of auditory hallucinations and perception of human speech in schizophrenia: A PET correlation study. Psychiatry Research: Neuroimaging, 122, 139-152.

Crow T.J. (2000). Schizophrenia as the price that Homo sapiens pays for language: a resolution of the central paradox in the origin of the species. Brain Research Reviews, 31, 118-129.

d'Alfonso, A.A.L., Aleman, A., Kessels, R.P.C., Schouten, E.A., Postma, A., van der Linden, J.A., Cahn, W., Greene, Y., de Haan, E.H.F. & Kahn, R.S. (2002). Transcranial magnetic stimulation of left auditory cortex in patients with schizophrenia: Effects on hallucinations and neurocognition. Journal of Neuropsychiatry and Clinical Neurosciences, 14, 77-79.

De Luca, M., Beckmann, C.F., De Stefano, N., Matthews, P.M. & Smith, S.M. (2005). fMRI resting state networks define distinct modes of long-distance interactions in the human brain. NeuroImage, in press.

David, A.S. (2004). The cognitive neuropsychiatry of auditory verbal hallucinations: An overview. Cognitive Neuropsychiatry, 9, 107-123.

Edmister, W.B., Talavage, T.M., Ledden, P.J. & Weisskoff, R.M. (1999). Improved auditory cortex imaging using clustered volume acquisitions, Human Brain Mapping, 7, 89–97.

Edwards J. (2001). Facial affect and affective prosody recognition in first-episode schizophrenia. Schizophrenia Research, 48, 235-253.

Ekman P., Friesen, W.V. (1976). Pictures of Facial Affect. In: Consulting Psychologists Press.

Hall, D.A., Haggard, M.P., Akeroyd, M.A., Palmer, A.R., Summerfield, A.Q., Elliott, M.R., Gurney, E.M. & Bowtell, R.W. (1999). "Sparse" temporal sampling in auditory fMRI, Human Brain Mapping, 7 , 213–223.

Hoffman, R.E., Gueorguieva, R., Hawkins, K.A., Varanko, M., Boutros, N.N., Wu, Y-T, Carroll, K. & Krystal, J.H. (2005). Temporoparietal transcranial magnetic stimulation for auditory hallucinations: Safety, efficacy and moderators in a fifty patient sample. Biological Psychiatry, 58, 97-104.

Hoffman, R.E., Hawkins, K.A., Gueorguieva, R., Boutros, N.N., Rachid, F., Carroll, K. & Krystal, J.H. (2003). Transcranial magnetic stimulation of left temporoparietal cortex and medication-resistant auditory hallucinations. Archives of General Psychiatry, 60, 49-56.

Keck, M.E., Engelman, M., Muller, M.B., Henniger, M.S., Hermann, B., Rupprecht, R., Neumann, I.D., Toschi, N., Landgraf, R. & Post, A. (2000). Repetitive transcranial magnetic stimulation induces active coping strategies and attenuates the

neuroendocrine stress response in rats. Journal of Psychiatric Research, 34, 265-276.

Lee K.H. (2004). Social cognition, brain networks and schizophrenia. Psychological Medicine,34, 391-400.

Lee, S-H, Kim, W., Chung, Y-C, Jung, K-H, Bahk, W-M, Jun, T-Y, Kim, K-S, George, M.S. & Chae, J-H. (2005). A doubel blind study showing that two weeks of daily repetitive TMS over the left or right temporoparietal cortex reduces symptoms in patients with schizophrenia who are having treatment-refractory auditory hallucinations. Neuroscience Letters, 376, 177-181.

Leentjens A., Wielaert S., van Harskamp F., Wilmink F. (1998). Disturbances of affective prosody in patients with schizophrenia; a cross sectional study. Journal of Neurology, Neurosurgery, and Psychiatry, 64, 375-378.

Lundqvist D., Flykt A. & Ohman, A. (1998). The Karolinska Directed Emotional Faces (KDEF). In: Department of Neurosciences, Karolinska Hospital, Stockholm.

Meyer M. (2002). FMRI reveals brain regions mediating slow prosodic modulations in spoken sentences. Human Brain Mapping, 17, 73-88.

McIntosh, A.M., Semple, D., Tasker, K., Harrison, L.K., Owens, D.G.C., Johnstone, E.C. & Ebmeier, K.P. (2004). Transcranial magnetic stimulation for auditory hallucinations in schizophrenia. Psychiatry Research, 127, 9-17.

Mitchell R.L. (2003). The neural response to emotional prosody, as revealed by functional magnetic resonance imaging. Neuropsychologia, 41, 1410-1421.

Moelker, A. & Pattynama, P.M.T. (2003). Acoustic noise concerns in functional magnetic resonance imaging, Human Brain Mapping, 20, 123–141.

Mukherjee, J., Christian, B.T., Narayanan, T.K., Shi, B. & Collins, D. (2005). Measurement of d-amphetamine-induced effects on the binding of dopamine D-2/D-3 receptor radioligand, ^{18}F-fallypride in extrastriatal brain regions in non-human primates using PET. Brain Research, 1032, 77-84.

Neggers, S.F., Langerak, T.R., Schutter, D.J., Mandl, R.C., Ramsey, N.F., Lemmens, P.J., Postma, A. (2004). A stereotactic method for image-guided transcranial magnetic stimulation validated with fMRI and motor-evoked potentials. NeuroImage, 21, 1805-1817.

Ohnishi, T., Hayashi, T., Okabe, S., Nonaka, I., Matsuda, H., Iida, H., Imabayashi, E., Watabe, H., Miyake, Y., Ogawa, M., Teramoto, N., Ohta, Y., Ejima, N., Sawada, T. & Ugawa, Y. (2004). Endogenous dopamine release induced by repetitive transcranial magnetic stimulation over the primary motor cortex: A [11] raclopride positron emission tomography study in anesthetized macaque monkeys. Biological Psychiatry, 55, 484-489.

Pascual-Leone A, Houser C, Reese K, Shotland L, Grafman J, Sato S, Valls-Solé J, Brasil-Neto J, Wassermann EM, Cohen L, Hallett M. (1993). Safety of rapid-rate transcranial magnetic stimulation in normal volunteers. Electroencephalography and Clinical Neurophysiology, 89, 120-130.

Pascual-Leone A., Walsh, v. & Rothwell, J. (2000). Transcranial magnetic stimulation in cognitive neuroscience--virtual lesion, chronometry, and functional connectivity. Current Opinion in Neurobiology, 10, 232-237.

Pinkham A.A.E.P.D.D. (2003). Implications for the neural basis of social cognition for the study of schizophrenia. The American Journal of Psychiatry, 160, 815-824.

Poldrack R.A., Wagner A.D., Prull M.W., Desmond J.E., Glover G.H. & Gabrieli J.D. (1999). Functional specialization for semantic and phonological processing in the left inferior prefrontal cortex. NeuroImage, 10, 15-35.

Poulet, E., Brunelin, J., Bediou, B., Bation, R., Forgeard, L., Dalery, J., d'Amato, T. & Saoud, M. (2005). Slow transcranial magnetic stimulation can rapidly reduce resistant auditory hallucinations in schizophrenia. Biological Psychiatry, 57, 188-191.

Price C.J., Moore C.J., Humphreys G.W., Wise R.S.J. (1997). Segregating semantic from phonological processes during reading. Journal of cognitive neuroscience, 9, 727-733.

Ross E.D., Orbelo D.M., Cartwright J., Hansel S., Burgard M., Testa J.A. & Buck R. (2001). Affective-prosodic deficits in schizophrenia: profiles of patients with brain damage and comparison with relation to schizophrenic symptoms. Journal of Neurology, Neurosurgery, and Psychiatry, 70, 597-604.

Sánchez-Gonzalez, M.A., García-Cabezas, M.A., Rico, B. & Cavada, C. (2005). The primate thalamus is a key target for brain dopamine. The journal of Neuroscience, 25, 6076-6083.

Schönfeldt-Lecuona, Grön, G., Walter, H., Büchler, N., Wunderlich, A., Spitzer, M. & Herwig, U. (2004). Stereotaxic rTMS for the treatment of auditory hallucinations in schizophrenia. NeuroReport, 15, 1669-1673.

Stewart L., Ellison, A., Walsh, V. & Cowey, A. (2001). The role of transcranial magnetic stimulation (TMS) in studies of vision, attention and cognition. Acta Psychologica, 107, 275-291.

Strafella, A.P., Paus, T., Barrett, J. & Dagher, A. (2001). Repetitive transcranial magnetic stimulation of the human prefrontal cortex induces dopamine release in the caudate nucleus. The Journal of Neuroscience, 21, RC157.

Strafella, A.P., Paus, T., Fraraccio, M & Dagher, A. (2003). Striatal dopamine release induced by repetitive transcranial magnetic stimulation of the human motor cortex. Brain, 126, 2609-2615.

Talvik, M., Nordstrom, A.L., Ollson, H., Halldin, C. & Farde, L. (2003). Decreased thalamic D2/D3 receptor binding in drug-naïve patients with schizophrenia: A PET study with [11C]FLB 457. International Journal of Neuropsychopharmacology, 6, 361-370.

Van de Ven, V.G., Formisano, E., Röder, C.H., Prvulovic, D., Bittner, R.A., Dietz, M.G., Hubl, D., Dierks, T., Federspiel, A., Esposito, F., Di Salle, F., Jansma, B., Goebel, R. & Lindenm D.E.J. (2005). The spatiotemporal pattern of auditory cortical responses during verbal hallucinations. NeuroImage, 27, 644-655.

Wasserman, E.W. (1997). Risk and safety of repetitive transcranial magnetic stimulation: report and suggested guidelines from the International Workshop on the Safety of Repetitive Transcranial Magnetic Stimulation, June 5-7, 1996. Electroencephalography and Clinical Neurophysiology, 108, 1-16.

Woodruff, P.W.R., Wright, I.C., Bullmore, E.T., Brammer, M., Howard, R.J., Williams, S.C.R., Shapleske, J., Rossell, S., David, A., McGuire, P.K. & Murray, R.M. (1997). Auditory hallucinations and the temporal cortical response to speech in

Bijlage 5: Flow chart

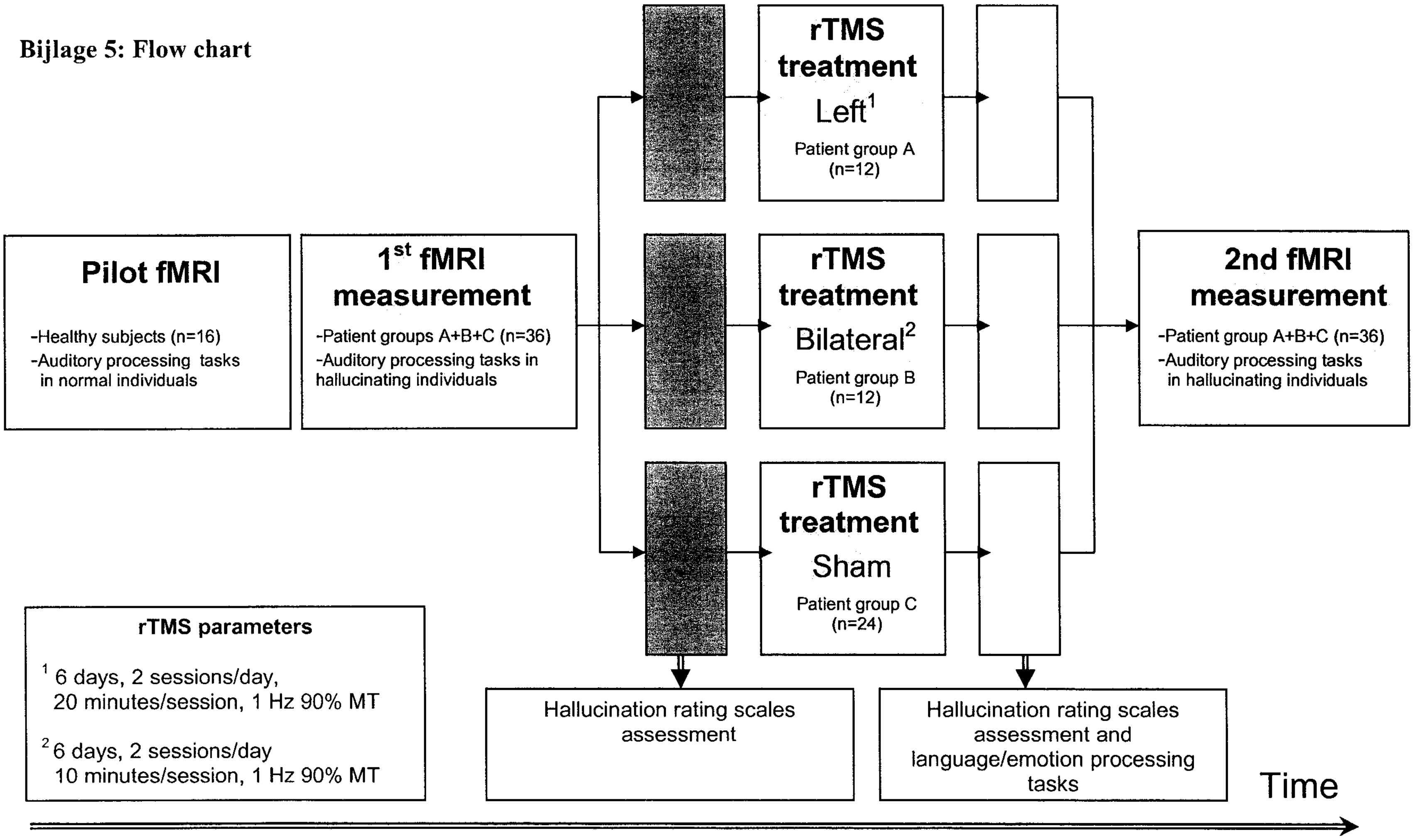

Supplement: Protocol S1 — The approved protocol of the study. (PDF) [file pone.0108828.s002.pdf]
